# The Evolving Facets of Bacterial Vaginosis: Implications for HIV Transmission

**DOI:** 10.1089/aid.2018.0304

**Published:** 2019-02-28

**Authors:** Lyle R. McKinnon, Sharon L. Achilles, Catriona S. Bradshaw, Adam Burgener, Tania Crucitti, David N. Fredricks, Heather B. Jaspan, Rupert Kaul, Charu Kaushic, Nichole Klatt, Douglas S. Kwon, Jeanne M. Marrazzo, Lindi Masson, R. Scott McClelland, Jacques Ravel, Janneke H.H.M. van de Wijgert, Lenka A. Vodstrcil, Gilda Tachedjian

**Affiliations:** ^1^ Department of Medical Microbiology and Infectious Diseases, University of Manitoba, Winnipeg, Canada.; ^2^ Centre for the AIDS Programme of Research in South Africa, Durban, South Africa.; ^3^ Department of Obstetrics, Gynecology, and Reproductive Sciences, University of Pittsburgh, Pittsburgh, Pennsylvania.; ^4^ Magee-Womens Research Institute, Pittsburgh, Pennsylvania.; ^5^ Central Clinical School, Monash University, Melbourne, Australia.; ^6^ Melbourne Sexual Health Centre, Alfred Hospital, Carlton, Australia.; ^7^ National HIV and Retrovirology Labs, Public Health Agency of Canada, Winnipeg, Canada.; ^8^ Departments of Obstetrics and Gynecology, and Medical Microbiology, University of Manitoba, Winnipeg, Canada.; ^9^ Department of Medicine Solna, Karolinska Institute, Stockholm, Sweden.; ^10^ Centre Pasteur du Cameroun, Yaoundé, Cameroon.; ^11^ Vaccine and Infectious Diseases, Fred Hutchinson Cancer Research Center, Seattle, Washington.; ^12^ Department of Medicine, University of Washington, Seattle, Washington.; ^13^ Seattle Children's Research Institute and University of Washington, Seattle, Washington.; ^14^ Department of Pathology, Division of Immunology, Institute of Infectious Disease and Molecular Medicine, University of Cape Town, Cape Town, South Africa.; ^15^ Department of Immunology, University of Toronto, Toronto, Canada.; ^16^ Department of Medicine, University of Toronto, Toronto, Canada.; ^17^ McMaster Immunology Research Centre, Michael G. DeGroote Centre for Learning and Discovery, McMaster University, Hamilton, Canada.; ^18^ Department of Pathology and Molecular Medicine, McMaster University, Hamilton, Canada.; ^19^ Department of Pediatrics, University of Miami, Miami, Florida.; ^20^ Ragon Institute of MGH, MIT, and Harvard, Massachusetts General Hospital, Cambridge, Massachusetts.; ^21^ Harvard Medical School, Boston, Massachusetts.; ^22^ Division of Infectious Disease, University of Alabama at Birmingham, Birmingham, Alabama.; ^23^ Division of Medical Virology, Institute of Infectious Disease and Molecular Medicine, University of Cape Town, Cape Town, South Africa.; ^24^ Centre for AIDS/HIV Program of Research in South Africa (CAPRISA) Centre of Excellence, University of Cape Town, Cape Town, South Africa.; ^25^ Department of Epidemiology, University of Washington, Seattle, Washington.; ^26^ Department of Global Health, University of Washington, Seattle, Washington.; ^27^ Institute for Genome Sciences and Department of Microbiology and Immunology, University of Maryland School of Medicine, Baltimore, Maryland.; ^28^ Julius Center for Health Sciences and Primary Care, University Medical Center Utrecht, Utrecht, the Netherlands.; ^29^ Institute of Infection and Global Health, University of Liverpool, Liverpool, United Kingdom.; ^30^ Disease Elimination Program, Life Sciences Discipline, Burnet Institute, Melbourne, Australia.; ^31^ Department of Microbiology, Monash University, Clayton, Australia.; ^32^ Department of Microbiology and Immunology at the Peter Doherty Institute for Infection and Immunity, The University of Melbourne, Melbourne, Australia.; ^33^ School of Science, College of Science, Engineering and Health, RMIT University, Melbourne, Australia.

**Keywords:** HIV, vaginal microbiota, bacterial vaginosis, HIV transmission, genital inflammation, female reproductive tract

## Abstract

Bacterial vaginosis (BV) is a common yet poorly understood vaginal condition that has become a major focus of HIV transmission and immunology research. Varied terminologies are used by clinicians and researchers to describe microbial communities that reside in the female reproductive tract (FRT), which is driven, in part, by microbial genetic and metabolic complexity, evolving diagnostic and molecular techniques, and multidisciplinary perspectives of clinicians, epidemiologists, microbiologists, and immunologists who all appreciate the scientific importance of understanding mechanisms that underlie BV. This Perspectives article aims to clarify the varied terms used to describe the cervicovaginal microbiota and its “nonoptimal” state, under the overarching term of BV. The ultimate goal is to move toward language standardization in future literature that facilitates a better understanding of the impact of BV on FRT immunology and risk of sexually transmitted infections, including HIV.

## Introduction

Bacteria are now recognized to play important immunological roles at all mucosal surfaces, and the female reproductive tract (FRT) is no exception.^1^ The entirety of “optimal” microbial communities associated with a mucosal site (i.e., the microbiota) is an important contributor to the effectiveness of the host mucosal barrier against infection.^2^ This is in contrast to “nonoptimal” microbial communities that are associated with the disruption of important physiological roles of bacteria at the mucosa.^1^ An example of nonoptimal microbiota is bacterial vaginosis (BV), a common vaginal condition in women of reproductive age associated with adverse urogenital and reproductive health outcomes, including an increased risk of HIV acquisition.^3–7^ BV affects 29% of women in the United States and 52% of women in sub-Saharan Africa, where HIV is also highly prevalent.^8^

BV is commonly diagnosed by clinicians using Amsel's criteria,^9^ defined here as “Amsel-BV,” a “vaginal discharge syndrome” wherein at least three out of four diagnostic criteria need to be met (Box 1). While women with BV can present with a vaginal discharge, BV is not typically associated with redness, swelling, or pain seen with “overt” inflammation,^10^ which is why it is referred to as “vaginosis” rather than “vaginitis.” However, BV is associated with “subclinical” genital inflammation, as determined by an increase in proinflammatory cytokines and chemokines^10–16^ associated with increased HIV risk.^17–19^ A second common method used to diagnose BV is by Nugent score, defined here as “Nugent-BV” (Box 1).^20^ The Nugent score captures bacterial morphotypes on a Gram stain, differentiating *Lactobacillus*-dominated bacterial communities from the presence of small Gram-variable rods (*Gardnerella vaginalis* morphotypes) and curved Gram-variable rods (*Mobiluncus* spp. morphotypes),^5^ which is an oversimplification of the actual ecology of BV.^21,22^

Nugent scoring has been used widely, particularly in epidemiology research, to define BV in large cohort studies, correlating BV to a wide range of adverse health outcomes.^18,19,23^ A proportion of women with Nugent-BV are clinically asymptomatic (“asymptomatic BV”). Nugent-BV can be sustained or transient, the latter representing a temporary shift in the vaginal microbiota mediated by intrinsic (menses) or extrinsic (sex) factors,^24^ which may or may not be associated with increased HIV risk. Some women with Amsel-BV may also not present with symptoms. While this may be uncommon for women presenting to a clinic, population-based Amsel screening will identify asymptomatic Amsel-BV-positive women. The presence of signs and symptoms of BV vary widely based on the perception of women and clinicians, complicating its diagnostic usefulness. Thus, BV that is diagnosed by either Amsel or Nugent methods can be further delineated as either “asymptomatic” or “symptomatic.”

While clinical manifestations of BV are important for patient care, it is now clear with advances in DNA sequencing technology that a broader range of nonoptimal cervicovaginal microbiota have relevance for adverse sexual and reproductive health outcomes. Cervicovaginal microbiota are genetically and ecologically complex, diverse, and dynamic.^24^ This combined with its health implications has made it a “hot topic” for molecular microbiologists. Several immunological and clinical associations of various cervicovaginal bacterial communities have now been characterized using molecular methods; these “nonoptimal” microbiota broadly overlap with BV defined by other methods, but are distinct, and we have termed these “Molecular-BV” (Box 1). As subtle differences between methods come to light, “Molecular-BV” should be further subdivided into terms that incorporate the specific molecular method (Box 1). One common method for microbiota characterization in recent literature is deep sequencing of the 16S rRNA gene. This method has been termed as a “broad-range PCR” method that measures the relative abundance of bacteria taxa without preconceived knowledge of the bacteria that are present.^25–28^ We propose that “nonoptimal” microbial communities defined by this technique be designated as “Seq-BV,” which would also incorporate whole-genome shotgun sequencing approaches. A second method is taxon-specific quantitative PCR (qPCR) that quantifies the absolute abundance of predetermined taxa,^26,29^ while not including others. We propose that “nonoptimal” taxa are designated as “qPCR-BV.” Metaproteomic analysis of cervicovaginal samples has also been employed to study the cervicovaginal environment, including bacterial composition, which has led to “optimal” and “nonoptimal” bacterial community classifications, where the latter could be designated as Prot-BV.^30–32^

The concept of “Molecular-BV,” as defined currently in research settings, is intended to be an “overarching” term to describe nonoptimal cervicovaginal microbiota characterized by molecular methods. This is not to suggest that it is not clinically relevant. An FDA-approved molecular diagnostic test for BV is being used in the United States.^33,34^ In addition, Molecular-BV has been associated with genital inflammation and/or adverse sexual and reproductive health outcomes, such as increased HIV risk,^17,29^ and therefore is prognostic for clinical outcomes. Understanding this distinction may enable better comparisons to studies where BV has been determined using Amsel's criteria or Nugent score.^18,19^

Molecular-BV bacterial communities are depleted of *Lactobacillus* spp., with a high relative abundance or load of facultative and/or obligate anaerobes (see Box 1 and Table 1 for microbial communities typical of Molecular-BV).^12,17,25–27^ These communities are usually “highly diverse” (i.e., high species richness or polymicrobial) and show “evenness” (i.e., not dominated by particular species), although they can be dominated by one species. Examples of Seq-BV include microbiota commonly referred to as cervicotypes 3 (CT3) and 4 (CT4), which proportionally are depleted of *Lactobacillus* spp. and predominately contain *G. vaginalis* or an increase in a mixture of diverse anaerobes comprising *Prevotella*, *Gardnerella*, *BVAB1*, *Sneathia*, and *Megasphera* spp., respectively.^11,17^ Examples of qPCR-BV include additional taxa shown to have a concentration-dependent association with genital inflammation and/or increased risk of HIV acquisition, for example, *Gemella asaccharolytica* and *Eggerthella* species type I.^29^

Box 1. **Proposed Definitions for Bacterial Vaginosis Based on Traditional Methods of Bacterial Vaginosis Diagnosis (Amsel and Nugent) and Molecular Techniques**

**Table:** 

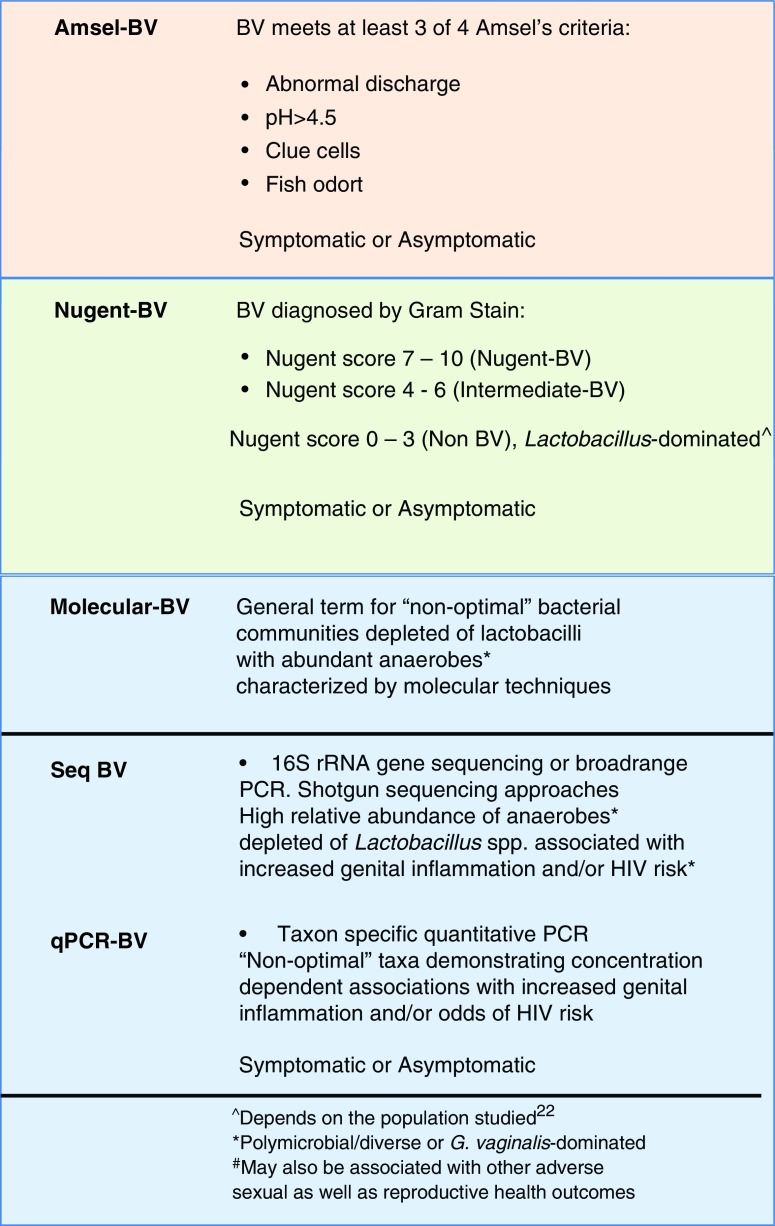

Color images are available online.

**Table 1.** Classification of Cervicovaginal Bacterial Communities Determined by 16S rRNA Gene Sequencing

*Abbreviation*	*Definition*	*Molecular-BV (Seq-BV)*
CST-I	*Lactobacillus crispatus*-dominated	No
CST-II	*Lactobacillus gasseri*-dominated	No
CST-III	*Lactobacillus iners*-dominated	No
CST-IVA	Modest *Lactobacillus* spp., higher relative abundance of facultative and/or obligate anaerobes, *BVAB1* and *Gardnerella vaginalis*	Yes
CST-IVB	Modest *Lactobacillus* spp., higher relative abundance of facultative and/or obligate anaerobes, *G. vaginalis* and *Atopobium vaginae*	Yes
CST-IVC	Lacking *Lactobacillus* spp. and more even in anaerobe composition (i.e., no bacteria dominate) comprising *Prevotella* among others, as well as *Anaerococcus*, *Finegoldia*, *Corynebacterium*, *Peptoniphilus*, *Megasphaera*, and *Gemella* spp.	Yes
CST-V	*Lactobacillus jensenii*-dominated	No
CT1	*L. crispatus*-dominated	No
CT2	*L. iners*-dominated	No
CT3	Depleted of *Lactobacillus* spp. and *G. vaginalis*-dominated	Yes
CT4	Depleted *Lactobacillus* spp. and polymicrobial with a higher relative abundance of facultative and/or obligate anaerobes comprising *Prevotella*, *Gardnerella*, *BVAB1*, *Sneathia*, and *Megasphera* spp.	Yes
C1	Depleted of *Lactobacillus* spp. and polymicrobial with a higher relative abundance of facultative and/or obligate anaerobes	Yes
C2	*L. crispatus*-dominated	No
C3	*L. iners*-dominated	No

C, compositional subtype^12,52^; CST, community state type^24,25^; CT, cervicotype^11,17^; Molecular-BV, bacterial vaginosis determined by characterizing vaginal or cervical samples using molecular methods; Seq-BV, BV as determined by 16S rRNA gene sequencing.

Techniques used to define Molecular-BV have demonstrated that an even larger proportion of asymptomatic women may be at risk of subclinical cervicovaginal inflammation and increased risk of acquiring sexually transmitted infections (STIs), including HIV.^17,29^ However, these overlapping yet distinct approaches for defining BV have led to some confusion among researchers in the field. A patchwork of terms describing BV and cervicovaginal microbiota continues to evolve as studies employ increasingly complex molecular measurements to better capture aspects of the microbiota that go beyond clinical or microscopic criteria by using bacterial relative or absolute bacterial abundances. This Perspectives article attempts to capture the heterogeneous terminology generated from this multidisciplinary research effort geared at understanding the intricate relationships between “BV,” as defined by Amsel, Nugent and molecular methods, cervicovaginal inflammation, and the risk of HIV/STIs.

**FIG. 1.**
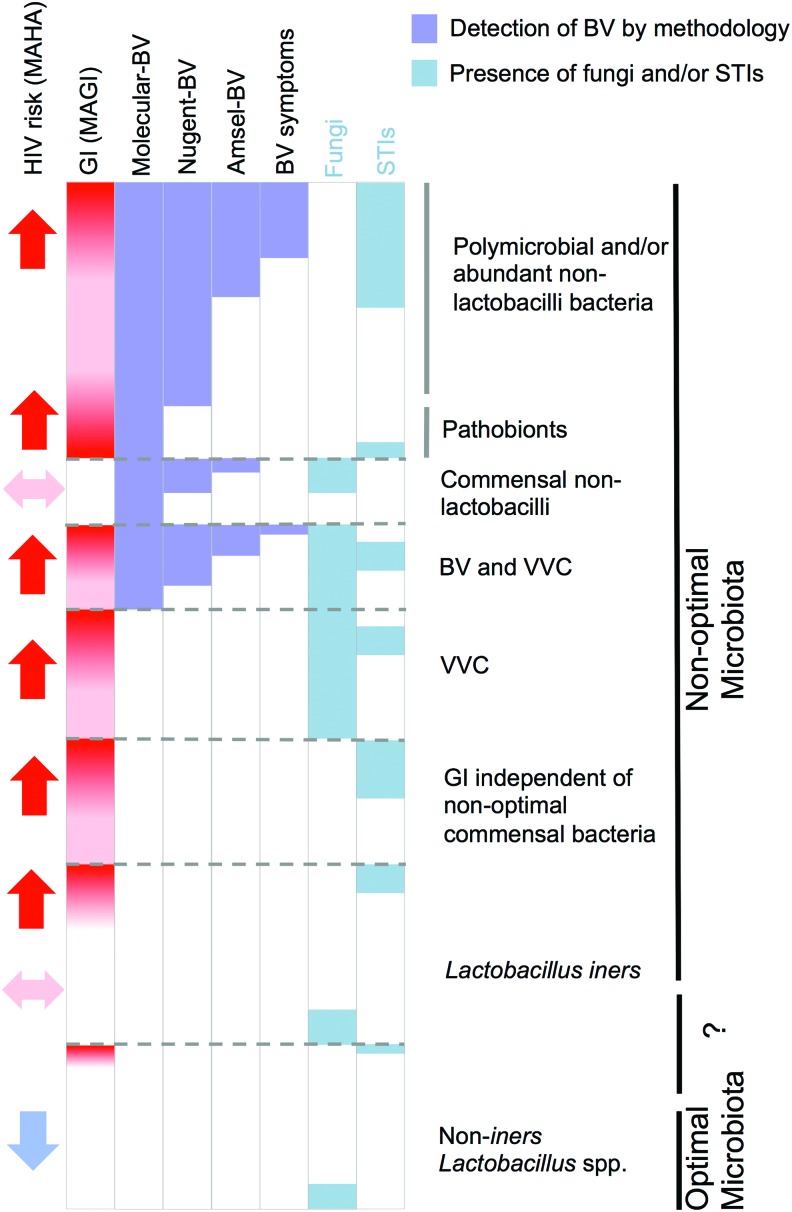
Microbial causes of genital inflammation and/or altered HIV susceptibility. Each microbial class can cause inflammation independently or in combination with other microorganisms that may also be present in the same women. Strategies to mitigate as many of these causes as possible may be key to achieving the optimal FRT mucosa associated with positive health outcomes, including protection against HIV infection. Optimal—cervicovaginal microbiota associated with no vaginal symptoms, lack of genital inflammation, and decreased HIV risk; nonoptimal—cervicovaginal microbiota associated with vaginal symptoms and/or genital inflammation and/or increased HIV risk. BV, bacterial vaginosis; FRT, female reproductive tract; GI, genital inflammation; MAGI, microbiota associated with genital inflammation; MAHA, microbiota associated with HIV acquisition; pathobionts, a symbiotic organism under normal circumstances that can become pathogenic, for example, *Proteobacteria*, *Streptococci*, *Staphylococci*, or *Enterococci* spp.; STIs, sexually transmitted infections; VVC, vulvovaginal candidiasis; *Lactobacillus* spp. (e.g., *Lactobacillus crispatus*) or strains that may not be optimal (e.g. *Lactobacillus iners*). Color images are available online.

While here we focus on BV, it is important to note that there are additional forms of “nonoptimal” cervicovaginal microbiota associated with vulvovaginal candidiasis (VVC) caused by *Candida* spp., and desquamative vaginitis or aerobic vaginitis caused by pathobionts *Proteobacteria*, *Streptococci*, *Staphylococci*, or *Enterococci* spp.^35,36^ These microbes and STIs other than HIV are clinically relevant and are associated with genital inflammation that can increase HIV risk (Fig. 1) and are, therefore, important when considering sources of inflammation in the cervicovaginal mucosa, but do not feature in the definitions of BV, which is the current focus of this Perspectives article.^35^ An update on this topic is planned in a report on the 2018 Keystone Symposia on the Role of the Genital Tract Microbiome in Sexual and Reproductive Health.

## Partial Overlap Between Amsel-BV, Nugent-BV, and Molecular-BV

**FIG. 2.**
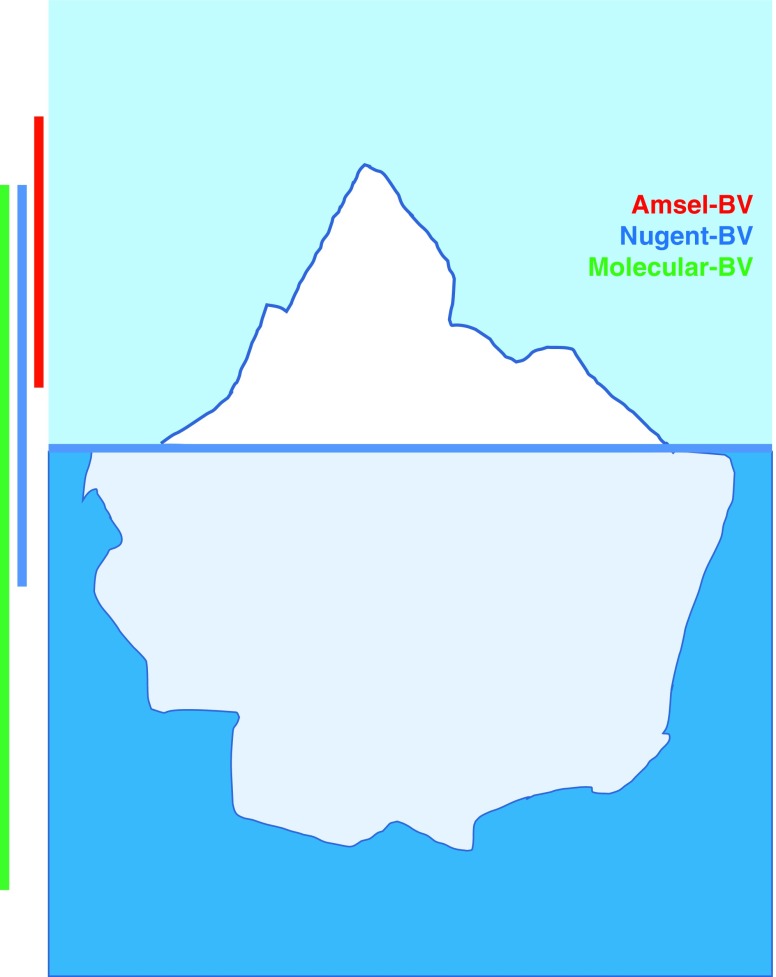
The “clinical iceberg” concept of adverse health outcomes, applied to BV. With better molecular methods, we now appreciate that clinically evident BV, as diagnosed by a technique such as Amsel's criteria (Amsel-BV), does not capture a high proportion of women diagnosed with BV by Nugent (Nugent-BV) or with molecular methods (Molecular-BV) that contributes to adverse sexual and reproductive health outcomes, including increased HIV risk. Not all Amsel-BV-positive samples are Nugent-BV- or Molecular-BV-positive. *Red vertical line* denotes Amsel-BV, *blue vertical line* denotes Nugent-BV, and *green vertical line* denotes Molecular-BV. Color images are available online.

Current evidence supports that only a minority of BV is symptomatic. Molecular-BV/Seq-BV, which categorizes microbiota into bacterial community types (Table 1), tends to correlate with vaginal pH and not with other Amsel criteria, such as clue cells and whiff test.^28,37^ Similarly, although Seq-BV correlates with Nugent-BV (Fig. 2), the overlap is incomplete.^17,22,25^ The majority of women who have an intermediate Nugent score (defined in Box 1) also have Seq-BV (e.g., CT3, CT4, and CST-IV),^11,17,22^ indicating an association with adverse health outcomes.^17^ We propose that data from primarily clinical (Amsel), microscopic (Nugent), and molecular evaluation of BV fit into an “iceberg” concept of a clinical/subclinical condition (Fig. 2). Amsel-BV is at the top of the iceberg, usually capturing clinically apparent nonoptimal vaginal microbiota, while both Nugent-BV and Molecular-BV include additional microbial states that can be subclinical (e.g., asymptomatic) but still clinically relevant for infection and/or health risk. It is worth noting that some Amsel-positive diagnoses may not be Nugent-positive due to either subjectivity of the Amsel criteria (e.g., vaginal discharge, odor) or perhaps differences in the ability of these tests to detect BV associated with biofilm versus planktonic BV, although these cases are uncommon.

## Nonoptimal Cervicovaginal Microbiota, Genital Inflammation, and HIV Acquisition Risk

Meta-analyses clearly demonstrate that women with Nugent-BV and/or Amsel-BV have an increased risk of acquiring HIV.^18,19^ Meta-analyses by Atashili *et al.* of 23 studies, including 30,739 women, reported a relative risk of 1.61 [95% confidence interval (CI) = 1.21–2.13] for HIV acquisition in women with Nugent-BV.^18^ Subsequently, an individual patient meta-analysis by Low *et al.* reported that Nugent-BV, measured at the seronegative visit before HIV diagnosis, was associated with an adjusted hazard ratio of 1.53 (95% CI = 1.24–1.89) for HIV acquisition risk.^19^ This study also demonstrated an elevated susceptibility to HIV (adjusted hazard ratio = 1.41; 95% CI = 1.12–1.79) in women with intermediate Nugent scores,^19^ suggesting that any Nugent score >3 may be a risk factor for HIV. On the basis of more recent molecular studies, a large proportion of these women would be expected to have Molecular-BV.^11,17,22^

Nugent-BV has repeatedly been associated with genital inflammation; in particular, proinflammatory cytokines are typically upregulated, whereas chemokines show no association, are upregulated (i.e., IL-8), or downregulated.^10,14–16^ This cytokine–chemokine distinction is likely due to the observation that BV is microbiologically multifaceted, and specific combinations of bacterial species may result in different host responses.^28^ The host response to the same bacterial communities could also vary between individuals, even though no studies have evaluated this specific question. In addition, some differences could be accounted for by methodological differences in sampling and measuring immune mediators in the genital tract.^10^

Molecular-BV has often been associated with both genital inflammation^11–13^ and an increased risk of HIV acquisition.^17,29^ A prospective study in South Africa reported that young women colonized with a highly diverse community (CT4) had a 4.4-fold (95% CI = 1.17–16.61) increased risk of acquiring HIV compared with women with *Lactobacillus crispatus-*dominant microbiota.^17^ Presence of *G. vaginalis*-dominated (CT3) cervicotype demonstrated a trend toward elevated HIV risk, although it did not reach statistical significance after adjusting for the presence of chlamydia.^17^ The *Lactobacillus iners*-dominated (CT2) cervicotype was not significantly associated with increased HIV risk.^17^ In this cohort, women with CT4 also had the greatest genital inflammation measured by levels of proinflammatory cytokines and chemokines, compared with women with *L. crispatus*-dominated microbiota, and followed by *G. vaginalis*-dominated and *L. iners*-dominated microbiota.^11^ Another nested case–control study in African women showed that vaginal bacterial diversity and several BV-associated bacterial species, including *Parvimonas* species types 1 and 2, *G. asaccharolytica*, *Mycoplasma hominis*, *Leptotrichia/Sneathia*, *Eggerthella* species type 1, and *Megasphaera* species, were significantly associated with higher risk of HIV acquisition.^29^

Several studies have demonstrated that elevated genital inflammation is associated with an increase in activated HIV target cells in the cervix,^11,17,38^ consistent with elevated HIV risk.^17^ However, not all studies have found an association between cervicovaginal bacterial communities and the frequency of CD4^+^ and CCR5^+^ activated or proliferating HIV target cells in the cervix,^12,39^ suggesting differences between geographic or ethnic populations. Alternatively, there could be other mechanisms by which nonoptimal cervicovaginal microbiota increase HIV risk, such as disruption of epithelial barrier integrity.^30–32,38^

## Effect Sizes for HIV Risk Determined by Nugent-BV/Amsel-BV Versus Molecular-BV

The effect sizes for Nugent-BV/Amsel-BV on HIV risk are typically smaller, that is, 60% increased risk^18,19^ compared with the effect size from Seq-BV on HIV risk (i.e., >4-fold).^17,28^ However, the latter was only from two studies, with modest number of women colonized with *L. crispatus*-dominated cervicovaginal microbiota, and needs to be confirmed.^17^ The large sample size (>30,000 individuals) evaluated in the BV meta-analysis could contribute to and explain the smaller effect sizes. The meta-analysis of Nugent-BV is also adjusted for potential confounders such as VVC and sexual behaviors, although these are incompletely controlled for in studies relying on Molecular-BV.^17,29^

While Nugent and Amsel are useful tools for epidemiological and clinical studies, they could be thought of as less sensitive, that is, underestimating the types of microbiota that put a woman at risk for HIV, compared with molecular evaluation of cervicovaginal microbiota, at least in research settings (Fig. 2). Nugent and molecular techniques can also detect brief episodes of “nonoptimal” microbiota (e.g., during menses)^24^ that may not cause significant genital inflammation and/or increase HIV risk, and it is likely that more broadly these communities may be dynamic, dependent upon a number of host and environmental factors (genital hygiene practices, sexual behavior, comorbidities, etc.). Therefore, the duration and frequency of “nonoptimal” vaginal microbiota are likely a critical factor requiring the incorporation of frequent sampling in longitudinal studies to better define the HIV risk associated with Nugent-BV and Molecular-BV.

## Not All *Lactobacillus s*pp. Are Associated with Reduced Genital Inflammation and Protection Against HIV Acquisition

*Lactobacillus* spp.-dominated cervicovaginal microbiota, and particularly with *L. crispatus*, are associated with a lack of genital inflammation relative to other bacterial communities.^11,12,16,17^ In a cross-sectional study, women with *L. crispatus*-dominated microbiota were less likely to be HIV positive compared with women with vaginal microbiota either dominated by *L. iners* or depleted of *Lactobacillus* spp.^40^ Furthermore, HIV was associated with a high bacterial load and abundance of strict and facultative anaerobes.^40^ While this cross-sectional analysis could be due to reverse causation (i.e., HIV could cause microbiome differences), this observation is supported by a prospective study in South African adolescent girls where *L. crispatus*-dominated cervicovaginal microbiota, but not *L. iners*, were associated with a decreased risk of acquiring HIV.^17^ In addition, *L. iners* was shown to be mildly inflammatory in *in vitro* cocultures with vaginal epithelial cells.^17^ Thus, while some *Lactobacillus* spp. are associated with decreased genital inflammation and HIV risk, not all *Lactobacillus* spp. are equally protective.

The difference in the ability of distinct *Lactobacillus* spp. to provide protection against HIV may be due to several factors that include their ability to produce lactic acid that is responsible for acidifying the vagina to a low pH.^25,41–43^ Lactic acid has been shown to have antimicrobial and immune modulatory properties.^44–47^ Modulation of inflammatory responses by *Lactobacillus* spp. may also be influenced by differences in cell wall properties between strains.^48,49^ Another factor is the apparent lower temporal stability of *L. iners*-dominated microbiota compared with *L. crispatus*-dominated microbiota. Indeed, when exposed to extrinsic and intrinsic factors, *L. iners-*dominated vaginal microbiota often transition to bacterial communities lacking *Lactobacillus* spp. and comprising a wide array of strict and facultative anaerobes.^24,50^ It is important to note that there is likely to be strain differences among *Lactobacillus* spp. (including *L. crispatus*) with levels of genital inflammation (Chetwin *et al.*, *Sci Reports*, in press) as well as *G. vaginalis* clades and HIV risk, which cannot be resolved by Nugent scoring or current 16S rRNA gene sequencing or qPCR approaches.

## Asymptomatic Nugent-BV or Molecular-BV Is Still Associated with Genital Inflammation

There is considerable controversy in the field regarding asymptomatic cases that lack *Lactobacillus* spp. as these appear disease free but may retain elevated risk of adverse health outcomes. Women who do not report any symptoms of BV but are positive for either Nugent-BV or Molecular-BV can still have “asymptomatic BV,”^35,51^ a state often associated with cervicovaginal microbiota dominated by *G. vaginalis* (e.g., CT3; Table 1)^17,24^ or are polymicrobial comprising facultative and/or obligate anaerobes while lacking *Lactobacillus* spp. often described as community state type IV (CST-IV),^25^ CT4,^17^ or compositional subtype 1 (C1)^12,52^ (Table 1). However, BV, including asymptomatic Molecular-BV or Nugent-BV, is often observed in African and Hispanic women,^17,25,28,51^ suggesting that genetic, socioeconomic, cultural, or behavioral factors might play a role alone or in combination. Furthermore, report of symptoms is subjective and varies among women since these may be “normal” if a woman has had them throughout her entire adult life. Yet her risk of HIV infection and other sexual and reproductive health outcomes may still be elevated due to asymptomatic Nugent-BV or Molecular-BV. In Gosmann *et al.*, the majority of women who acquired HIV were asymptomatic and negative for Nugent-BV, despite having evidence of Molecular-BV.^17^ Thus, reliance on symptoms alone is not recommended for assessing increased risk for HIV. In the future, if methods to positively and effectively alter the microbiota are achieved, screening asymptomatic women in clinical practice may also be appropriate.

## Terminology, Definitions, and Recommendations

We list terminologies often used in the cervicovaginal microbiome field and provide definitions as a guide for investigators to promote precision and consistency (Box 1, Tables 1 and 2). We also propose the following recommendations for the field to consider.

1. There is a preferred consensus developing around the term “optimal” to describe cervicovaginal microbiota often associated with favorable health outcomes and characterized by a lack of symptoms, dominance of non-*L*. *iners Lactobacillus* spp., and a lack of genital inflammation. “Nonoptimal” is preferred to describe microbiota associated with adverse sexual and reproductive health outcomes, including increased HIV acquisition risk (Fig. 1).
2. Avoid use of the terms “dysbiotic” or “abnormal” microbiota, since both of these terms imply divergence from a normal state that might not exist for all women. For women with asymptomatic BV and low levels of genital inflammation, their microbial community might represent their “normal” microbial state, and these terms may inappropriately stigmatize these women.
3. We suggest that descriptive terms to describe the microbiota (i.e., in recommendations 1 and 2) be tested for acceptability with women in qualitative studies such that terminology is friendly to women who may be likely to benefit from the development of approaches to reverse the consequences of Molecular-BV.
4. We propose new terminology that specifies the method used to diagnose BV—that is, Amsel-BV (based on Amsel criteria), Nugent-BV (defined by Nugent score), and Molecular-BV (based on molecular methods)—with subcategories defining the molecular technique employed, that is, Seq-BV or qPCR-BV (Box 1). Appropriate abbreviations could also be used for “nonoptimal” microbiota identified through new and emerging technologies, including metagenomics, transcriptomics, metabolomics, and metaproteomics.
5. Amsel-BV, Nugent-BV, and Molecular-BV can be further delineated into symptomatic or asymptomatic. Studies based on stratification of symptomatic BV is not recommended, given that “symptoms” can be subjective and do not fully capture the cervicovaginal microbiota associated with important health outcomes.
6. Not all “nonoptimal” microbiota are “highly diverse,” that is, *G. vaginalis-*dominated microbiota (e.g., CT3, CST-IVB),^11,17,24,25^ which has also been referred to as “low-diversity anaerobic dysbiosis” as distinct from “high-diversity anaerobic dysbiosis,” such as CT4 and CST-IVC.^35^ However, it is important to be precise when using the term “diverse” to describe microbiota. The use of the term “diverse” can be ambiguous with respect to 16S rRNA gene sequencing data. It is often used to describe communities such as CT4 and CST-IVC that have “species richness,” that is, many different species in a microbial ecosystem and “evenness,” that is, not dominated by particular species. However, it is possible that a community dominated by *L. crispatus* (e.g., CT1, CST-I, C2) can have high within-community intraspecies diversity (Ravel, unpublished). In addition, “*L. crispatus*-dominated” microbiota could also be diverse, as a result of diversity due to very low abundance taxa representing <1% of the community (i.e., an uneven community).
7. When describing *Lactobacillus* spp. as “optimal” or “beneficial,” specify the *Lactobacillus* species. Not all *Lactobacillus* spp. or strains make “optimal” cervicovaginal microbiota. Current data indicate that most strains of *L. iners* are less stable,^24^ associated with increased genital inflammation,^17^ and encode factors that may be harmful to the vaginal mucosa.^53,54^
8. We propose terminology that describes cervicovaginal microbiota associated with genital inflammation (MAGI) and microbiota associated with HIV acquisition (MAHA). While the focus of this Perspectives article is on BV, these terms would also encompass STIs, pathobionts, and VVC. Use of these terminologies, including “susceptible” for HIV, requires that there is evidence that the cervicovaginal microbiota increase genital inflammation (MAGI) and/or HIV risk (MAHA). These are overlapping but distinct microbiota-associated phenotypes (Fig. 1).
9. There is a need for standardization of methodology and terminology for characterizing bacterial communities by 16S rRNA gene sequencing (e.g., CSTs, CTs, and Cs) defined by clustering analysis preferably compared with a reference database comprising a large number of cervicovaginal microbiota to avoid collapsing of distinct clusters due to low number of samples being analyzed. Such a database (data from 12,000 samples) has been established by Jacques Ravel, which will be made available for use (unpublished). Other areas of standardization are sample site (e.g., vaginal, cervical, and lavage), sample processing, and the use of primers directed at the same 16S rRNA gene region for amplification.
10. More frequent sampling of cervicovaginal microbiota is recommended when determining the association of a cervicovaginal microbiota state with adverse health outcomes as well as more rigorous controlling of confounders that are associated with genital inflammation, including STIs and VVC.
12. Advance scientific knowledge into the mechanisms that underpin epidemiological associations observed with distinct microbial communities and HIV risk that is critical for driving the development of viable treatment and prevention modalities to promote an optimal microbiota and prevent HIV. Develop better tissue and animal models that recapitulate the FRT and can be colonized with women's cervicovaginal microbiota and infected with HIV.

**Table 2.** Descriptive Terms for Cervicovaginal Microbiota

	*Definition*
**Terms for optimal microbiota**	
Optimal	Microbiota associated with no vaginal symptoms, lack of genital inflammation and favorable sexual and reproductive health outcomes, including decreased risk of HIV acquisition
Eubiosis	Microbiota that are “optimal”
Healthy	Microbiota that are “optimal”
Normal	Often used to describe “optimal” microbiota; less preferred terminology since “normal” is difficult to define
*Lactobacillus*-dominant	Microbiota dominated by *Lactobacillus* spp. usually determined by 16S rRNA gene sequencing
Beneficial lactobacilli	Optimal *Lactobacillus* spp., often used to distinguish *L. crispatus* (optimal) from *L. iners*
Protective	Microbiota that protects against adverse health outcomes such as HIV; evidence of *in vivo* protection is required
Non-BV	Microbiota composed of bacteria not consistent with BV
Microflora	Outdated terminology that should not be used to describe microbiota; suggests that microbiota are composed of plants rather than bacteria, fungi, viruses, archaea, and protists
**Terms for nonoptimal microbiota**	
Nonoptimal	Microbiota associated with vaginal symptoms, and/or genital inflammation and/or adverse sexual and reproductive health outcomes, including increased risk of HIV acquisition
Dysbiosis	Imbalance in the microbiota or impaired microbiota or “nonoptimal” microbiota; avoid using this terminology for women with asymptomatic BV and low levels of genital inflammation as their microbiota might represent their “normal” state and may inappropriately be stigmatizing
Low-diversity anaerobic dysbiosis	*G. vaginalis-* or *A. vaginae*-dominated microbiota associated with adverse sexual and reproductive health outcomes
High-diversity anaerobic dysbiosis	Polymicrobial community depleted of *Lactobacillus* spp. associated with adverse sexual and reproductive health outcomes
Harmful	Less preferred terminology for microbiota associated with vaginal symptoms, genital inflammation, and/or an increased risk of adverse sexual and reproductive health outcomes
Non-*Lactobacillus*-dominant	Non-*Lactobacillus* spp.-dominated bacterial community
Polymicrobial	Multiple bacterial species usually depleted of *Lactobacillus* spp. with an increase in obligate and/or facultative anaerobes
Diverse	Used to describe microbial communities comprising multiple bacterial species in the ecosystem; needs to be defined since meaning can be ambiguous with respect to 16S rRNA gene sequencing
Susceptible	Microbiota associated with increased risk of HIV and other STIs or adverse reproductive health outcomes; requires evidence to link microbiota with adverse health outcomes
MAGI	Microbiome associated with genital inflammation; this term can also encompass STIs and other microbes associated with genital inflammation, including *Candida* spp.; requires evidence linking microbiota with genital inflammation
MAHA	Microbiome associated with HIV acquisition; this term can also encompass STIs and other microbes associated with genital inflammation, including *Candida* spp.; requires evidence linking microbiota with increased HIV risk
Pathobionts	Symbiotic organism under normal circumstances that becomes pathogenic, e.g., *Proteobacteria*, *Streptococci*, *Staphylococci*, or *Enterococci* spp.

BV, bacterial vaginosis; MAGI, microbiota associated with genital inflammation; MAHA, microbiota associated with HIV acquisition; STIs, sexually transmitted infections.

## Conclusions

Regardless of how it is defined, it is clear that BV is a topic of growing interest and importance for sexual and reproductive health in women. To facilitate making sense of this expanding research effort, we propose using standardized definitions that “best capture genital inflammation and/or HIV/STI risk.” On this basis, molecular methods for characterizing the cervicovaginal microbiota are anticipated to replace both Nugent and Amsel as a BV gold standard. This does not imply that Nugent and Amsel no longer have a role in assessing clinical BV. In clinical practice, Amsel will remain useful for diagnosing symptomatic BV; however, new sensitive and specific molecular diagnostic tests are becoming available, such as the FDA-approved BD MAX vaginal panel.^33,34^ Many properly trained sites may opt to continue using Nugent-BV due to cost or logistical reasons, since there is a plethora of data published on Nugent-BV, and it is known to capture a proportion of individuals colonized with abundance of non-*Lactobacillus*-dominated bacterial communities with high specificity. However, to really understand the role of the nonoptimal cervicovaginal microbiota in HIV and inflammation, it will be necessary to employ a range of “omic” techniques, including metagenomics (next-generation DNA sequencing of whole bacteria, not only the 16S rRNA gene), transcriptomics, proteomics, and metabolomics, in conjunction with immunological measurements. Use of these techniques will be necessary to advance our knowledge of BV and conditions that promote BV so that better treatments can be developed and to stop the cycle of frequent recurrence that is commonplace with current treatments.
